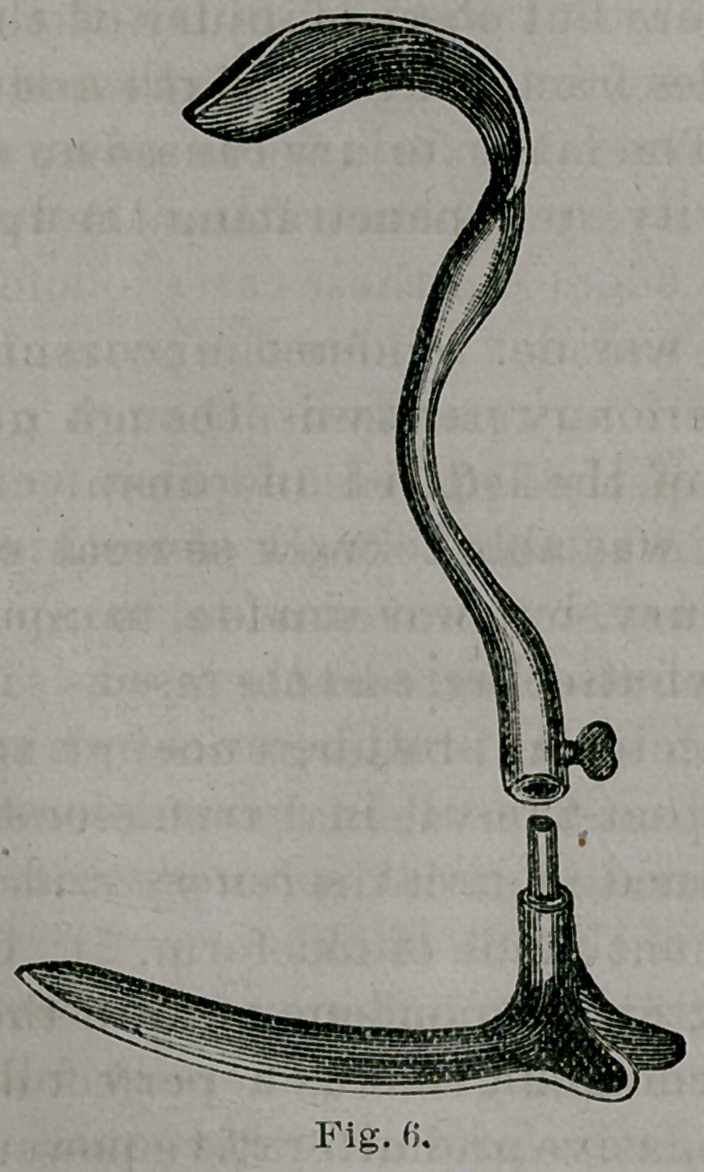# The Application of Pressure in Diseases of the Uterus, Ovaries and Peri-Uterine Structures

**Published:** 1882-09

**Authors:** V. H. Taliaferro

**Affiliations:** Atlanta, Ga.; Professor of Obstetrics and Diseases of Women and Children in the Atlanta Medical College


ATLANTA
### MEDICAL REGISTER.

VOL. I.] SEPTEMBER, 1882. [NO. 12

### Original.

### THE APPLICATION OF PRESSURE IN DISEASES OF THE UTERUS, OVARIES AND PERI-UTERINE STRUCTURES.

BY V. H. TALIAFERRO, M D., ATLANTA, GA.
Professor of Obstetrics and Dise ses of Women and Children in the Atlanta Medical College.

At the annual meeting of the Medical Association of Georgia, held in Atlanta in April, 1878, I submitted a paper upon "The Application of Pressure in Diseases of the Uterus." This subject was designed to embrace those disorders of the peri-uterine structures so frequently, and I might say constantly, co-existing with diseases of the uterus. It was shown in the detail of cases that the congestive, hypertrophic and hyperplastic affections of that organ were curable by the *pressure of the tampon.* It was also shown that the inflammatory deposits and adhesions so often co-existing and seriously complicating those disorders, and which hitherto had been regarded as incurable (save in exceptional cases by time), were equally amenable to the same simple method of treatment. Of the four cases referred to, illustrative of this new method of treatment, two had retroflexion of the uterus with *deposits of lymph and adhesions. The application of pressure* by the tampon in these cases was made not to remedy the displacements (for this was impossible in consequence of the fixation by adhesions), but to relieve the congestion and disordered local nutrition, and to destroy by disintegration and absorption the deposits of lymph and to make admissible the use of the pessary if needed. This was successfully accomplished in two and four months, and the displacements subsequently rectified by the Albert Smith pessary. The adhesions yielded in these cases in less than the usual time. I rarely promise cure under four or six months; and in old cases where the deposits are extensive it will often take a very much longer time of continuous and persistent treatment. I have yet to encounter a single case of this nature that failed of steady and continuous improvement from the beginning of treatment and of final cure.

Usually in these cases of inflammatory deposit there is a subacute or chronic pelvic peritonitis, with a marked tendency to recurrent acute attacks. In such conditions the life of the patient is threatened with each recurrent attack, either by extension of the serous inflammation, or cellular abscess, or both, as is most usually the case. This tendency to recurrence is promptly arrested by the tamponade, and the patient in the majority of cases speedily relieved of her necessary confinement to room and bed.

The treatment of pelvic adhesions, by forcible elongation and rupture, advocated by Dr. Van DeWarker, of Syracuse, N. Y., at the late meeting of the American Gynecological Society, was very properly condemned in the discussion which followed the reading of the paper. The only safety of the patient from hazardons consequences in these attempts at forcible rupture, is the facility with which the anterior rectal wall yields to the force applied, permitting the elevation *en masse* of rectum, uterus and deposits. The only safe and rational methods of treatment in these pelvic deposits and adhesions when chronic, are by the *hot water vaginal douche* of Emmet, and *pressure by the tampon*, as taught by myself. As to the comparative merits of these two methods there can be no question. True, when the inflammation is *acute* the hot water is preferable and the tampon contraindicated, but when the inflammation is chronic, and organized lymph has *fixed* the pelvic organs in an adherent mass, then *the tampon* is the remedy *par excellence.* Indeed it is the only remedy by which we can as yet hope to free the organs from their imprisoned adhesions. If the tampon was applicable alone to this class of cases it would be a priceless boon to our thereapeutic resources, but as we shall see it covers a large field in uterine pathology—a pathology in which congestion and its sequences stand pre-eminent.

Dr. Van De Warker very truthfully states in the outset of his paper, already referred to, " that there was probably no other disease* peculiar to women which offers a darker outlook to the patient than that to which his paper referred. Dr. Sims said : " Such cases were very difficult to cure, and very few were cured." Dr. Bozeman said : " For many years he had been engaged in treating this class of cases, uncomplicated with fistulæ, by *columnizing* the vagina with carbolized cotton, a practice which he brought out in a paper presented to the society in 1878, and again in 1879."

Dr. Bozeman's paper of 1878, to which he refers, was upon " Retroversion and prolapsus of the uterus in relation to the simple lacerations of the cervix uteri and their treatment by bloody operations," read before the American Gynecological Society at its meeting held in Philadelphia September 25th, 26th and 27th of that year. In this paper, presented *five months subsequent to my own*, Dr. Bozeman condemns what he is pleased to term *Emmet's bloody operation on the cervix*, and considers his, (Emmet's), preparatory treatment of cervical rupture with prolapsus and retroversion, by means of the hot water vaginal douche and air distended elastic and ring pessaries, as inefficient, and presents a method which he claims as his own, and which he seems to think quite sufficient to take the place of Emmet's preparatory treatment and bloody operations on the cervix. This *wonderful* method offered as a substitute for one of the most eminently successful and valuable operations in uterine surgery is: First. *The knee chest position*, which Dr. Sims taught him while they both lived in Montgomery, Ala., some thirty years ago.

*Uterine adhesions.

Second. The distension of the vagina to its fullest extent by the admission of air, as taught him by Dr. Sims about the same time. Third. A *flat*, *thin* narrow column of car-

Fig. 1.—Showing normal position of the pelvic organs, to be able the better to appreciate the principles here taught.

bolized cotton or wool extending from the posterior vaginal cul-de-sac to just within the pubic arch and range of the perineum, to "support not only the uterus and walls of the vagina, but also the ovaries, which are so frequently prolapsed in these cases." The tampon as a support to the uterus was taught him by Dr. Barker in 1853, and subsequently by Dr. Thomas in his work on "Diseases of Women," and still later by myself in "The application of pressure in diseases of the uterus." These are the *discoveries* for which priority is claimed, and which are to supercede pessaries and bloody operations. Nowhere in his paper of 1878 is allusion made to the so-called column of cotton as a remedy for *pelvic deposits or uterine adhesions*, and yet in the late discussion of the Gynecological Society on the "Forcible rupture of uterine adhesions," by Dr. Van De Warker, he (Bozeman) informs us that "for many years he had been engaged in treating this class of cases uncomplicated with fistulæ, by *columnizing the vagina* with carbolized cotton, a practice which *he brought out* in a paper presented to the society in 1878 and again in 1879."

In Dr. Munde's paper, read before the American Gynæcological Society, in 1879, after detailing my own method of tamponing as a valuable measure, where the parts are too tender to bear a pessary, he says: "This method of packing the vagina was first recommended in print by Taliaferro, of Georgia, for cases of cellulitis, metritis and oophoritis and displacements in which a pessary cannot be borne; but Dr. Bozeman, I am informed, claims the priority of the principle. It certainly is an excellent measure, and particularly applicable in those cases where hyperplastic or retroversion of a hyperesthetic uterus accompany prolapsus of the ovaries. The steady pressure of the tamponade in itself is a potent agent in the reduction of the inflammatory congestion, œdema of the pelvic organs, and frequently gives relief, even though the prolapsed ovary remains unreplaced." In the discussion which follows, Dr. Bozeman very *modestly* accepts what Dr. Munde says somebody told him, and he even *borrows* my sheeps wool and iodoform ointment which I taught him the use of in my paper upon "The application of pressure in diseases of the uterus," in April, 1878.

The first mention ever made by Dr. Bozeman of his *column of cotton* was in September, 1878, at the meeting of the American Gynæcological Society, and then it was proposed only as a remedy for displacements of the uterus. My paper upon *pressure by the tampon* was presented to the Medical Association of Georgia at its annual meeting, held in Atlanta, April, 1878, being five months prior to the publication of Dr. Bozeman, and yet he claims priority for the principle of the tampon, both as a therapeutic and mechanical resource.

If the Doctor seeks to appear in the pitiable attitude of appropriating the labors of others, and making claims which he fails to substantiate, then he must abide the just indignation to be awarded him.

It may not be uninteresting to see how closely the Doctor followed me before the American Gynæcological Society, five months after reading my paper, which he received from my own hands through mail.

He says: "First. To aid in accomplishing what is desired, the knee-elbow or knee-chest position is of the greatest importance. When the patient is placed in this position, we have the most complete extension of the vertebral column possible, the highest degree of relaxation of the diaphragm and the abdominal muscles; and at the same time the pelvic and abdominal viscera fall forward.

"Second. The vagina must be distended to its fullest extent by the admission of air and the use of a suitable speculum.

"Third. There must be formed a firm pyramidal column of carbolized cotton or wool extending from the posterior vaginal cul-de-sac obliquely downward across the axis of the vagina to a point just within the pubic arch and the range of the perineum. * * * * * The pieces of cotton *or wool* with which the column is formed may be secured in loops of strong sewing-thread, so that the patient can remove them at the end of two or three days, and take a vaginal douche of warm water preparatory to a renewal of the procedure. When the above indications are all fulfilled, the woman assumes the position upon her feet and goes about her daily business, whatever this may be."

In my paper five months prior, and which Dr. Bozeman read, I state: "In applying the packing, it should be borne in mind that the vaginal canal *must be distended and elongated* to its utmost capacity, and the uterus must occupy *its utmost degree of elevation* in the pelvis. These can be obtained only *in the knee-chest position*, so accurately described by Dr. Sims." * * * * * * In this changed condition of things, we find the vagina presenting in shape somewhat that of an irregular cone, with its base above and apex below; and in this changed condition, with the uterus *hanging from the vaginal vault from one to two inches higher* than its normal position (see Fig. 2), the tampon is applied, filling completely and compactly the entire vagina.

Fig. 2

The first one or two pieces of the tampon to go immediately over the cervix should be *cotton*, well filled with the best quality of glycerine. The cotton holds glycerine better than the wool. These little pieces should contain as much glycerine as they can be made to hold, by placing the cotton in the palm of the hand and rubbing in the glycerine, little by little, until it is a pulpy mass. These may be placed with the dressing-forceps immediately upon the cervix, or one behind it and the other in front. The pledgets of wool are thus successfully applied dry, each one being first rolled upon itself rather tightly in order to give the requisite firmness and solidity to the packing. The vault of the vagina is first well filled and the packing proceeded with carefully, the pledgets rolled upon themselves, being placed here and there, and packed with probe or dressing-forceps; all parts of the vagina being packed as equally firm as possible, and yet not so solid at any point as to cause discomfort. The vaginal canal is thus filled down to the muscular floor of the pelvis, but not below it. If the vaginal outlet is distended it will give discomfort, and a portion of the tampon may be lost. The vaginal orifice should close over the filling." (See Fig 3.)

Fig. 3.

It will be seen how closely the Doctor followed me in the details, while the reading of my paper was fresh in his mind. He modifies my tampon by reducing greatly its size (calling it *columnizing* for tamponing), and applying a very loose and thin packing, which is worthless as a means of support or pressure.

Prior to the publication of my paper, the Doctor had used the cotton for stretching and preparing the vagina for operation in vesico-vaginal fistula; but that he had used it for other purposes, he has signally failed to show.

Dr. Bozeman calls the attention of Dr. Munde to his failure to give him credit in his *Journal of Obstetrics* for priority in the use of the tampon as a therapeutic resource. How his priority is recognized, I quote from Dr. Munde's "Minor Surgical Gynæcology" as follows:

"As a mechanical support and stimulus to the pelvic vessels and as an alterative to the pelvic tissues by means of direct pressure it exerts on them." Says: "This method was first systematically described in print by Dr. V. H. Taliaferro, of Atlanta, Georgia, but Dr. Bozeman, of New

York, claims to have used it many years previously. According to Dr. Taliaferro (whose description I follow, as it was from him I first learned the details of the practice), the solid packing of the vagina with cotton and wool is an excellent remedy in subinvolution, areolar hyperplasia, desensus, and other dislocations of the uterus; chronic pelvic pontinitis and cellulitis, adhesions (and I would add), chronic ovaritis."

I trust I will be pardoned for the space and time consumed in substantiating my claims, and if I have been a little severe, I can safely say I have been just. I am sure I shall not be censured for protecting my labors from the hand of the despoiler. If the literary thief is despicable, what must be said of the man of science who purloins the brain work of his professional brother? It is, alas! the supreme culmination of ignominy.

The therapeutic action of pressure by the tampon may be thus classified: 1st. It diminishes blood supply and nutrition. 2d. It increases absorption. 3d. It destroys hyperplastic tissue by retrograde metamorphoses. 4th. It diminishes nerve activity. 5th. It rectifies displacements. In comparison with other methods of treatment I claim for pressure by the tampon: 1st. More immediate and rapid results. 2d. Patients are not confined to bed, but on the contrary, the pressure affords them such relief as enables them to walk about in pursuit of their ordinary employment and pleasures, while by the ordinary measures of caustic, sponge tents, etc., a more or less prolonged rest and confinement to bed is necessary. 3d. *The suspension of sexual connection*, a therapeutic auxilary of importance and one usually beyond control by the ordinary methods of treatment. 4th. Security from the inflammatory accidents incident to caustics, curette and tents. 5th. Softening and dilatation of the indurated tissues, while caustics and curette toughen and contract them. 6th. The integrity and vitality of the parts are maintained, while by caustics and curette there is a sacrifice of normal structure, often damaging and irreparable.

In all uterine congestions we have excessive blood supply and nutrition. Pressure diminishes these, and hence its application to a large class of uterine disorders. In subinvolution, in displacements, in endometritis, in cervical rupture, it is the accompanying congestion and increased nerve activity which gives rise to the pain, the distant sympathies and nervous anguish. Nothing so certainly illustrates this fact as the rapid disappearance of these symptoms by the application of pressure. In the supernutrition of old congestions so often found in subinvolution of the uterus, the common result is proliferation of connective tissue, as we have it in areolar hyperplasia, or the chronic metritis of older writers. This condition has been for all time the opprobrium of gynecologists. It is here that the entire armamenta of caustics, from the most powerful to the mildest, have done their best, and left only wounds and scars and irreparable damage. It is here that the tamponade is most happy in its results—promptly lessening the blood supply and restoring the nutrition of the tissues; destroying by disintegration and absorption the adventitious structures, and restoring the enlarged and indurated organ to its normal size and condition.

Fig. 4.

In very old cases of this kind where the tissues have become thoroughly hardened, and the progress of cure is found to be slow, we often extend the pressure to the uterine cavity by means of the cloth tent (see Fig. 4) to the cavity of the uterus in conjunction with the vaginal tamponade. Upon each renewal of the tampon an additional tent may be used, until as many as five or six are introduced at one time. In this way we get thorough dilatation and softening of the uterine structures without confinement to bed and without discomfort to the patient. Of course, where tents of any kind are used we must be sure that no contra-indication exists in the way of perimetritis or peri-uterine deposits, or great tenderness of the uterus. These must all be thoroughly removed before tents are admissible.

In cervical rupture, complicated as it so invariably is with congestion and excessive nerve activity, the pressure of the tamponade affords us a simple and pleasant remedy for the rapid restoration of the organ to a healthy condition preparatory to operation. In a case of extensive bilateral rupture of the cervix recently operated on at my private infirmary the cervix was thoroughly everted and covered with angry granular erosions. In this case, as in others, I touched the eroded surface thoroughly every few days with sulphate of copper. Usually the erosions disappeared by the pressure with the disappearance of the congestion, but when they are obstinate the blue stone is my favorite remedy. Where the diseased condition of the mucous membrane extends to the cavity, and that structure breaks down in fungous degeneration, the blue stone is an effective and valuable adjunct to the tampon in the restoration of the mucous membrane to its normal healthy state. In using it, first dilate the cervical canal with the cloth tent as directed; wash out the uterus with warm water, and then with forceps carry a suitable piece of solid blue stone in the cavity and apply it to every part. One or two applications will usually be sufficient. In using the remedy in this way some years ago upon a patient preparatory for operation for extensive bilateral cervical rupture, the piece of blue stone of some size dropped from the forceps in the cavity of the uterus, and I was unable to clasp it again in the instrument. The blue stone is a painful remedy and I was very fearful as to the immediate result. The patient lived about two and a half miles from my office, and after applying a light tamponade, the first pledgets saturated with glycerine, I sent her home in her carriage, with instructions to take to her bed upon arrival and send for me if she had any considerable pain. At my visit the next day I found to my surprise that she had gotten along with tolerable comfort. Upon removal of the dressing I found it thoroughly colored with the blue stone, and with no unpleasant effects upon the vagina. From this simple application the fungous granulations and menorrhagia which had been so troublesome and a complication in this case, disappeared to return no more.

I am convinced that in a very large proportion of cases of the so-called *endometritis*, granular erosions and fungous degeneration of the endometrium, congestion is the prime pathological factor. The terminal venules and arterioles of the uterine blood-vessels form a dense and delicate network upon the uterine mucosa, and hence in old congestions of the uterus the mucous membrane so often breaks down in fungous degeneration with its distressing and troublesome sequelæ.

How often, indeed, do we find granular erosion of the os extending into the cervical canal without involvement of the nabothian glands. The thick tenacious mucus which plugs the os in cervical endometritis is pathognomonic of glandular inflammation. The cervical erosion of the os uteri and fungous degeneration of the endometrium often exists independent of inflammation. The softening and breaking down of the mucous membrane is the result simply of long continued congestion and malnutrition. Acting upon this pathology as well as from the bad results following them, I have well-nigh ceased the use of caustics and the curette.

In threatened abortions, whether occurring from displacements, congestions, cervical rupture or the so-called irritable uterus, the tampon is a valuable resource. I am quite sure I have been able to carry a number of cases to term by this means that must otherwise have miscarried. Some of these cases were of the habitual variety—the most intractable, perhaps, of all others.

In several cases of inveterate nausea and vomiting with great exhaustion, and confinement to bed for weeks, the relief afforded by the tamponade was almost immediate, the patients being soon out of bed with good appetites, eating well and retaining the food.

The irritable bladder which so frequently complicates the uterine trouble, is often the first symptom to disappear upon the application of pressure. Where the uterus is adherent to the rectum or the cervix is drawn back by shortening of the utero-sacral ligaments with the posterior wall of the bladder necessarily dragged backward, an irritable bladder, with frequent and painful urination, is the common result. In these cases I know of no treatment comparable to the pressure.

In chronic inflammation, congestion and enlargement of the ovaries, the pressure acts as promptly and efficiently as in congestion of the uterus. In a number of cases of this nature I have been agreeably disappointed by the happy results.

I have used the tampon in a number of cases of fibroid tumors of the uterus, and while I cannot in any case report cure, I can in every case report decided improvement both in the general health and the local affection. Usually the menorrhagia and the metrorrhagia have been promptly controlled and the menstrual interval made complete. When the menstrual hemorrhage is considerable I am in the habit of keeping up the tampon through the entire menstrual period. The tamponade when made with glycerine and iodoform, directly to be described, quite sufficiently controls the hemorrhage, and makes a thoroughly antiseptic dressing. If, as occurs in some very obstinate cases, the hemorrhage is not sufficiently lessened to make it approximate in quantity the normal flow, then the glycerine may be left off and iodoform and tannin used. The tampon in these cases should be renewed daily, but I have several times, from providential causes, left them for five or six days, without detriment to the patient, and when removed found to be perfectly free from odor. While pressure is being made by the tampon, if the patient be required to encase herself in a long and tightly-fitting corset, and at the same time slowly but thoroughly saturate her system with ergot, the worst of these cases will steadily and surely improve.

*Original Method of Applying the Tampon.*—My original method of using the tampon for the application of pressure in diseases of the uterine organs, appears in a paper presented to the Medical Association of Georgia, convened in Atlanta in April, 1878, and is thus described: "In applying the packing, it should always be borne in mind that the vaginal canal *must be distended and elongated* to its utmost capacity; and the uterus must occupy its utmost degree of elevation in the pelvis. These can be obtained only *in the knee chest position* so accurately described by Dr. Sims.

"The material for the tampon should be of *sheep's wool.* Its elasticity and porosity especially fit it for this purpose. It should be clean and carded into bats and properly disinfected with carbolic acid. The essential pre-requisites, then, for the packing are: *the position of the woman*, Sims' speculum, dressing forceps and sheep's wool.

"Before a good direct light, across the bed, upon a good hard mattress, or better upon a table, the patient is placed upon her *knees and chest*, with the knees *directly* under the hips and a little separated. The thighs should be *perpendicular and at right angles with the table.* "She must not arch the spine upward, for this brings into forcible action the abdominal muscle, which should be perfectly relaxed, with the spine curved downward, as we see it in sway-backed animals. With these precautions fully impressed upon her, she is to breathe easily and relax the muscles of the abdomen" (Sims). Dress-strings and corsets should be removed and loosened completely. Many women, usually short ones, are unable to bring the chest flat upon the table, but they can "bend the body forward until the head is brought down to the plane of the table, where it must rest in the two hands, its weight supported on the left parietal bone, while the *elbows are thrown widely out from the sides*" (Sims.)* The *outstretched elbows* bring the chest as nearly as possible always upon the table. These details may appear tediously careful, but without an attentive observance of them the tampon will fail in its objects. The carded wool bats should be broken into small pledgets, or separate pieces, the patient in position as described, the perineum elevated with a short and broad blade Sims speculum, and we are ready for the packing. The first one or two pieces of the tampon to go immediately over the cervix should be *cotton*, well filled with the best quality of glycerine. The cotton holds the glycerine better than the wool. These little pieces should contain as much glycerine as they can be made to hold, by placing the cotton in the palm of the hand and rubbing in the glycerine little by little until it is a pulpy mass. These may be placed, with the dressing forceps, immediately upon the

*Italics mine.

cervix, or one behind it and the other in front. The pledgets of wool are then applied dry, each one being successively rolled upon itself rather tightly in order to give the requisite firmness and solidity to the packing. The vault of the vagina is first well filled and the packing proceeded with *carefully*, the pledgets rolled upon themselves being placed here and there, and packed with dressing forceps: all parts of the vagina being packed as equally firm as possible, and yet not too solid at any point for comfort. The vaginal canal is thus filled down to the muscular floor of the pelvis, but not below it. If the vaginal outlet is distended it will give discomfort, and a portion of the tampon may be lost. The vaginal orifice should close over the filling."

This method of packing the vagina is illustrated in Fig. 3.

I have long since discarded the sheep's wool for cotton, which I originally used. I was induced to do this because of the superior convenience of the cotton, and because it can be made more compact, and hence a greater degree of pressure obtained. Instead of extending the tampon from the vault of the vagina to its floor, I now rarely extend it further than the upper third of the vagina, and often not more than the upper fourth. In case an extra degree of pressure is desired, the upper half or still more rarely the upper two-thirds of the vagina is packed. In the large majority of cases of congestion, displacements and adhesions, the tampon is made to occupy only the upper fourth of the vagina. (See Fig. 5.) If the tenderness of the organs admits it, this partial tampon should be *very firm.* If there is considerable tenderness the dressing should be very light, and the pressure gradually increased as the tenderness subsides, until the packing is made as firm and compact as it can be made. When *pressure* is desired, a loose packing is not sufficient, and the so-called columns of Bozeman are worthless. This column is simply a loose, flat tampon extending from the posterior cul-de-sac to the ostium vaginæ. It can neither give *support* or *pressure* to the uterine organs. Its value consists mainly in separating and possibly softening the vaginal walls, for which it was used by its author until he read my paper in the spring of 1878.

Fig. 5.

The tampon applied only to the upper third of the vagina does not interfere with the bladder or rectum by its pressure upon those organs. The lower portion of the vagina closes in the tampon which occupies its vault. The tampon is thus securely held in place and rests upon the elastic column formed by the approximated vaginal walls. See Fig. 5.

The advantages of the tampon thus applied are: 1st. It does not reach the urethra nor make uncomfortable pressure upon the bladder and rectum. It answers all the purposes of pressure of the more extensive tampon. 3d. It does not interrupt the physiological mobility of the uterus. 4th. It keeps in place more securely than the more extensive tampon.

In witnessing the application of the tampon by my medical friends, some of whom are familiar with uterine manipulations, I have been astonished at the *awkward* manner in which the operation is made. Almost invaimated. Such a tampon utterly fails in its objects, and is worn by the patient with great discomfort. The packing should always be tight and firm in the vaginal vault, and if this is properly done no advantage is gained by filling the lower part of the vagina.

The size of the uterus should be closely watched during treatment by the tampon. If it is continued after the uterus has been reduced to its normal size *atrophy* of that organ, with deficient menstruation, will result. I have had several cases of this kind, where the uterus in the beginning was abnormally large, and have been obliged to restore the size of the organ, with its impaired function, by the use of the stem pessary. How long should the tampon remain in place? For the first few weeks of treatment the renewal should be made every two days, and subsequently every three days. I have often left the tampon for six or seven days, and several times, from providential causes, have permitted it to remain for twelve days, and once for four weeks, and in every instance found it upon removal perfectly free from odor. In these cases iodoform was a constituent of the tamponade. Where the patient is accustomed to the tampon its long retention does not give discomfort if it has been properly applied and disinfected. As an antiseptic and disinfectant I know of nothing equal to iodoform. I have never removed a foul tamponade when iodoform was one of the ingredients used. The first pledget of cotton, which should be large enough to spread over the cervix and in part the vaginal roof, (see Fig. 5) should be thoroughly saturated with the *best glycerine* and then spread with an ointment made of iodoform, ʒi; balsam Peru, ʒii; vaseline cerate, ℥i. The balsam of Peru thoroughly deodorizes the iodoform and gives us a perfect disinfectant. Its disinfectant, sedative and alterative powers render it peculiarly valuable as a uterine remedy. It is quite fusible and quickly absorbed into the surrounding structures, upon which it exerts its stimulant and alterative powers. In inflammatory deposits and adhesions I regard it as especially serviceable as an adjunct to the tampon. It was recommended as a local remedy in my paper upon "The Application of Pressure in Diseases of the Uterus," published in 1878, and I do not know of its having been used in uterine therapeutics prior to this date. In some patients when the system becomes saturated with the remedy, it causes an unpleasant nervousness, and occasionally I have had to suspend it or use it in reduced quantities. Tannin is often serviceable used in conjunction with the glycerine and iodoform. Where the uterine organs and peri-uterine structures are excessively tender a poultice made of flax seed meal, glycerine, tannin and iodoform, gives great comfort and rapidly improves the condition of the parts. The poultice is easily applied upon pledgets of cotton over the entire vaginal roof and held securely in place with the tampon, which occupies the upper vagina.

The perineal elevator seen in place in Fig. 2 is better represented in Fig. 6. The blade is flat and thin, like that

Fig. 6.

of Nott's speculum, and flanged at the proximal end to separate and hold apart the nates. The handle terminates in a curve to fit the hand, and is light and more convenient for tamponing than an ordinary Sims' speculum.

The drawings for the cuts with which this paper is illustrated were made by my friend and associate, Dr. G. H. Noble, and the engraving was done by our Atlanta artist, Mr. E. H. Hyde.

## Figures and Tables

**Fig. 1. f1:**
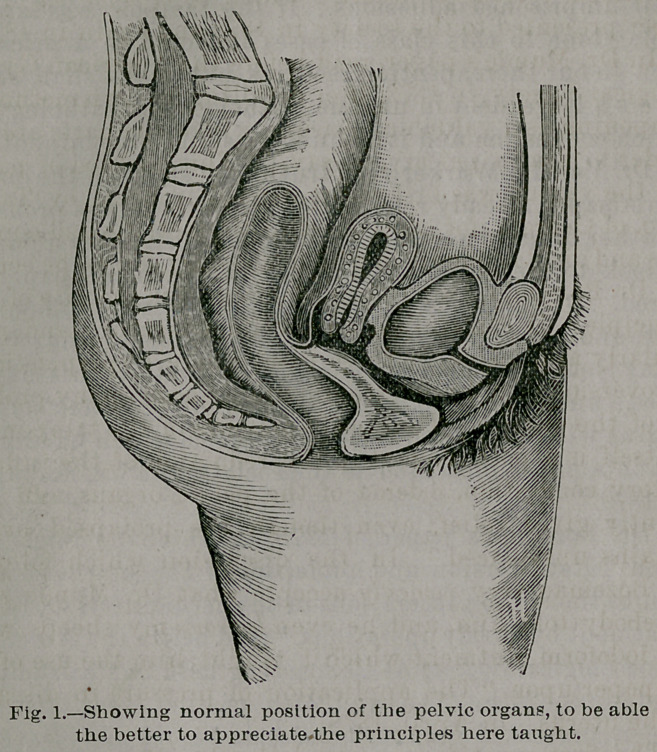


**Fig. 2. f2:**
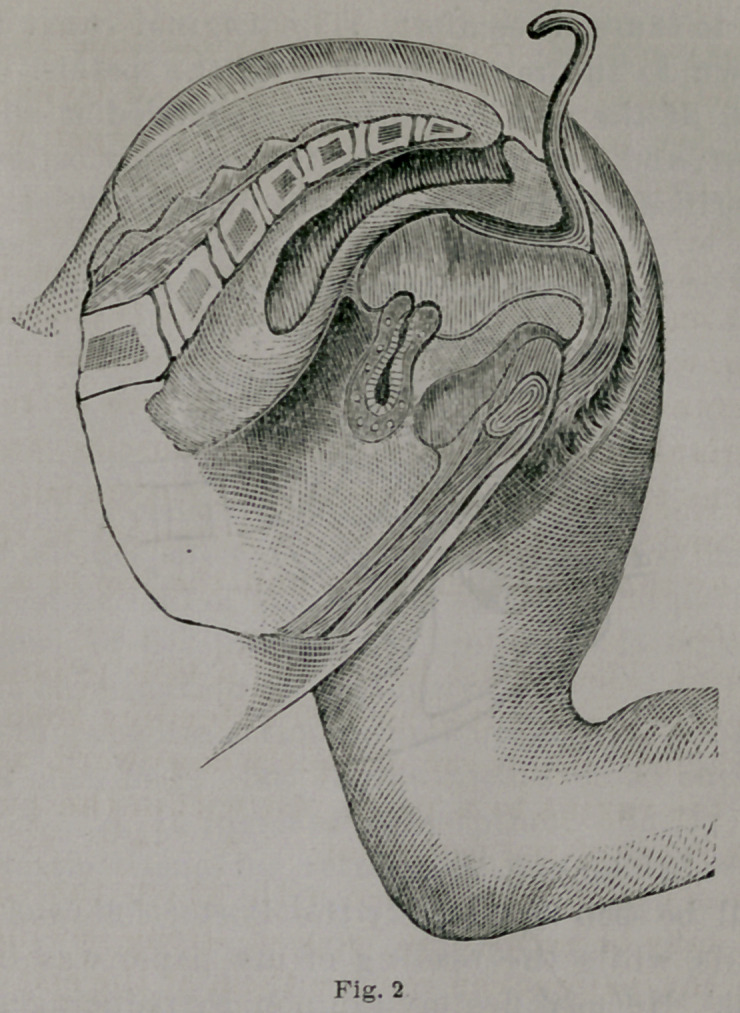


**Fig. 3. f3:**
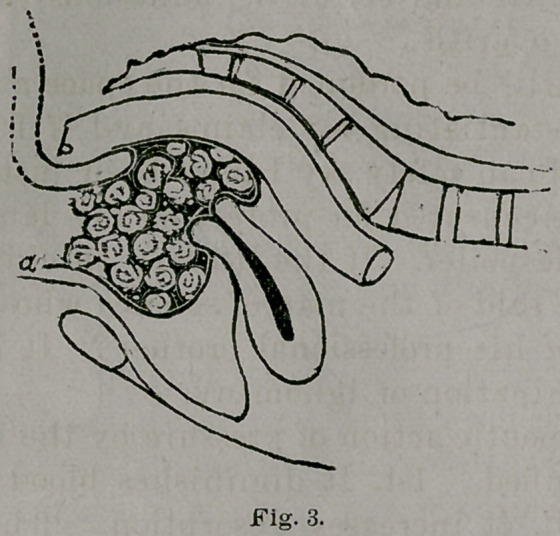


**Fig. 4. f4:**
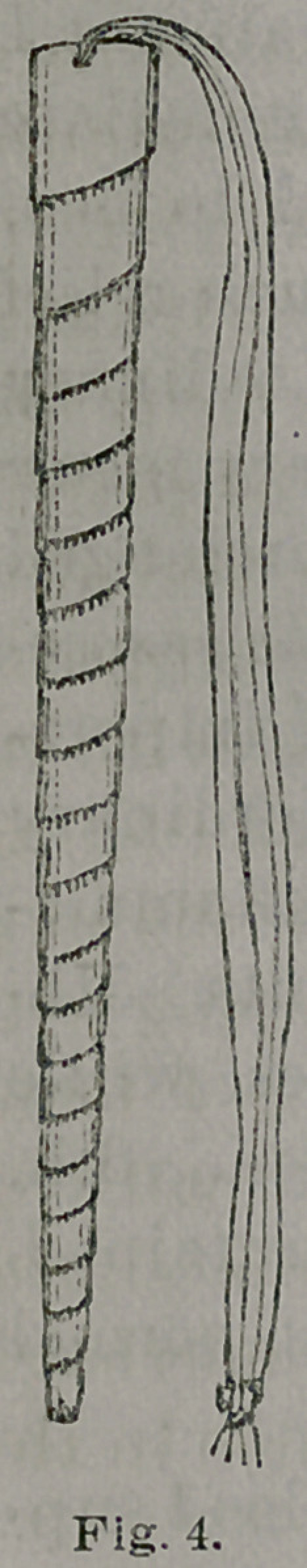


**Fig. 5. f5:**
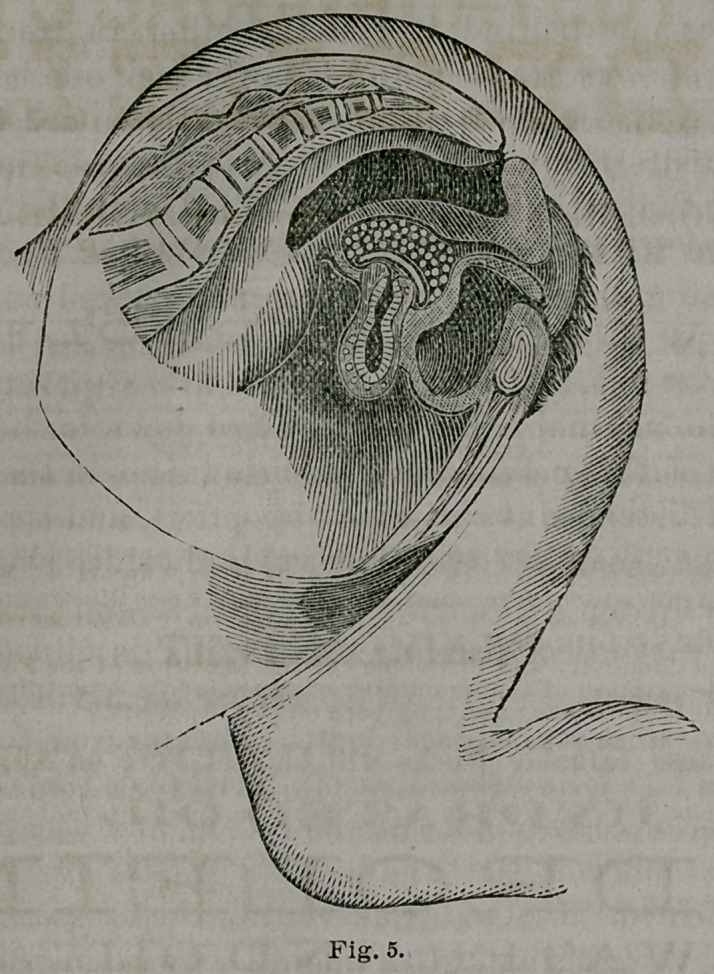


**Fig. 6. f6:**